# Intra-procedural Cone-Beam Computed Tomography for Ultrasound-Guided Microwave Ablation of Hepatocellular Carcinoma: An Initial Experience

**DOI:** 10.7759/cureus.102262

**Published:** 2026-01-25

**Authors:** Salma Kamaledeen, Jake Cowen, Christopher Ball

**Affiliations:** 1 Clinical Radiology, Portsmouth Hospitals University NHS Trust, Portsmouth, GBR

**Keywords:** cone-beam computed tomography (cbct), hepatocellular carcinoma (hcc), interventional oncology, percutaneous microwave ablation, ultrasound-guided

## Abstract

Ultrasound-guided microwave ablation is an alternative to the more widespread computed tomography (CT)-guided ablation. It confers some advantages such as immediate probe position adjustments, observation of real-time effects of ablation, lower radiation dose, and reduction in procedure time without increasing demand on diagnostic CT scanners. However, opponents of this technique cite the difficulty of visualising the thermal probe position or ablation zone. Incorporating cone-beam CT (CBCT) during ultrasound-guided tumour ablation allows improved visualisation and accuracy of thermal probe placement, but little data is available on this combined approach. We describe our centre's experience of the use of intra-procedural CBCT to aid the accuracy of ultrasound-guided microwave tumour ablation of hepatocellular carcinoma (HCC) in the liver. Our results demonstrate that employing intra-procedural CBCT during ultrasound-guided ablation of HCC liver tumours can be a safe and effective minimally invasive technique.

## Introduction

Ultrasound (US)-guided microwave ablation (MWA) of liver tumours has largely superseded radiofrequency ablation (RFA) as a minimally invasive treatment modality for liver tumours offering comparable efficacy to surgical resection with fewer associated complications and shorter recovery period [1]. This technique involves the precise placement of microwave probes into liver tumours under real-time US guidance, followed by the delivery of high-frequency microwave energy to induce tumour necrosis [2]. While US provides excellent real-time visualisation during the procedure, its ability to accurately assess the ablation zone and detect residual tumour tissue post-treatment is limited, potentially leading to incomplete tumour ablation and tumour recurrence.

Cone-beam computed tomography (CBCT), also known as Dyna-CT (dynamic volume computed tomography) and C-arm CBCT, is an imaging modality that combines the advantages of both CT and fluoroscopy, providing high-resolution three-dimensional images in real-time during the interventional procedure [3]. By offering detailed anatomical information and the ability to visualise the ablation zone and adjacent structures with improved accuracy and spatial resolution, CBCT may optimise the planning, execution, and assessment of MWA procedures. 

The integration of CBCT with US guidance offers several potential advantages. It enables precise needle placement and the real-time monitoring of the ablation process, minimising the risk of damage to critical structures such as blood vessels and bile ducts whilst ensuring complete tumour coverage. It provides immediate feedback on the extent of tumour ablation, allowing for prompt adjustments to optimise treatment outcomes and reduce the likelihood of tumour recurrence [4]. It facilitates the detection of residual tumour tissue post-ablation, enabling early identification and targeted re-treatment, if necessary, thereby improving long-term oncological outcomes [5,6]. Lastly, in the context of increased imaging demand within radiology departments, it frees the diagnostic CT scanner by allowing procedures to be performed in the interventional radiology (IR) or "Angio" suite.

In this study, we evaluate the feasibility and technical success of intra-procedural CBCT in assisting US-guided MWA of hepatocellular carcinoma (HCC) at our centre.

## Materials and methods

Patient and tumour characteristics

A retrospective review of US-guided microwave liver tumour MWA performed with intra-procedural CBCT to aid thermal probe placement between 2021 and 2023 was carried out at Queen Alexandra Hospital in Portsmouth, UK, using our Picture Archiving and Communications System (PACS), Computerised Radiology Information System (CRIS), and digital patient records.We examined the demographic data, number and segmental location of tumours, tumour size, and recurrence. IRB approval was not required.

Statistical analysis

A Shapiro-Wilk test of normal distribution was performed for the continuous variables (age, largest lesion size). The mean and standard deviation were calculated.

Procedure technique

HCC ablations at our centre are performed in the IR suite by a single consultant abdominal and non-vascular interventional radiologist with expertise in image-guided ablation of liver and lung lesions. All patients are admitted into the IR Day Case Unit on the day of the procedure which is performed under general anaesthesia.

**Figure 1:**
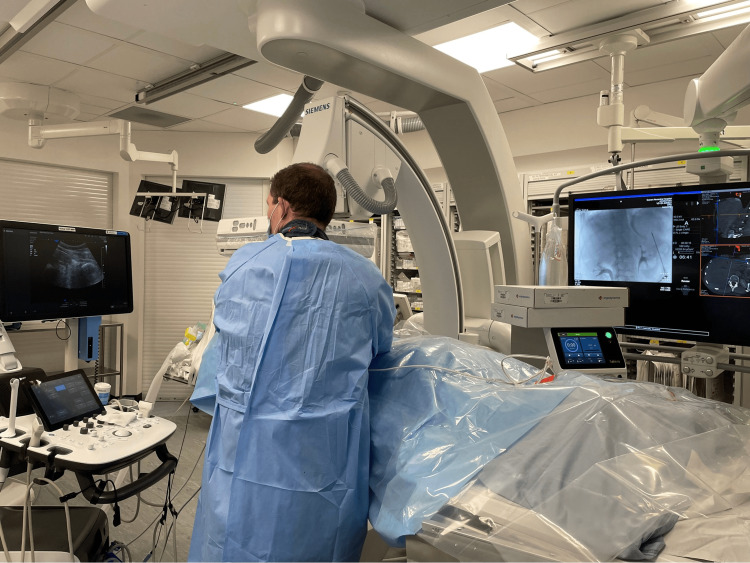
Interventional radiology suite arrangement The anaesthetist stands at the head of the patient, the radiologist is on the right of the patient, the ultrasound machine is in front of the operator, and the thermal ablation machine and screens are opposite the operator. The nurse stands on the left of the patient, and the radiographer stands at the foot of the patient.

Figure 1 shows the typical arrangement and the position of the radiologist in relation to the patient, US machine, and C-arm. Consent is in two stages, with patients consenting on the phone days prior to the procedure if not already done so in clinic and in-person on the morning of the procedure by the anaesthetist and radiologist.

Pre-operative diagnostic contrast-enhanced multi-detector CT (MDCT) is used for pre-procedure planning. During the procedure, the lesion is located by US, and probe placement commences under US visualisation. We use the Angiodynamics Solero Microwave Tissue Ablation System (Latham, New York, United States) with a single needle applicator in 14 cm, 19 cm, or 29 cm length depending on lesion depth. CBCT with or without intravenous contrast is performed to delineate the thermal probe position in relation to the centre of the lesion. Contrast used is 90 mls of Omnipaque 240 (GE Healthcare, Chicago, Illinois, United States) injected intravenously at 3 ml/s at 100% concentration with 55 second injection delay and breath hold. CBCT is performed without magnification at 90 kV and mA depending on patient size. Images were reconstructed using a standard convolution kernel.

Both US and Dyna-CT images are used for the correlation of optimal thermal probe placement in the centre of the lesion. All our cases are performed using the free-hand technique. A three-minute thermal ablation at 140 kw is applied per lesion. Dyna-CT demarcates the lesion within the ablation zone to ensure it is adequately ablated. If multiple liver lesions are present, these are ablated sequentially in the same session. Track ablation is performed at the end of the procedure at 100 kw for 20 seconds.

The patient is admitted overnight with post-operative analgesia and discharged within 24 hours. Patients are scanned at three months post-ablation as standard with conventional CT and then at six months and 12 months with CT or MRI, followed by six-monthly surveillance US for HCC. Technical success is defined as complete coverage of the tumour with an adequate safety margin on CBCT and three-month CT.

**Figure 2:**
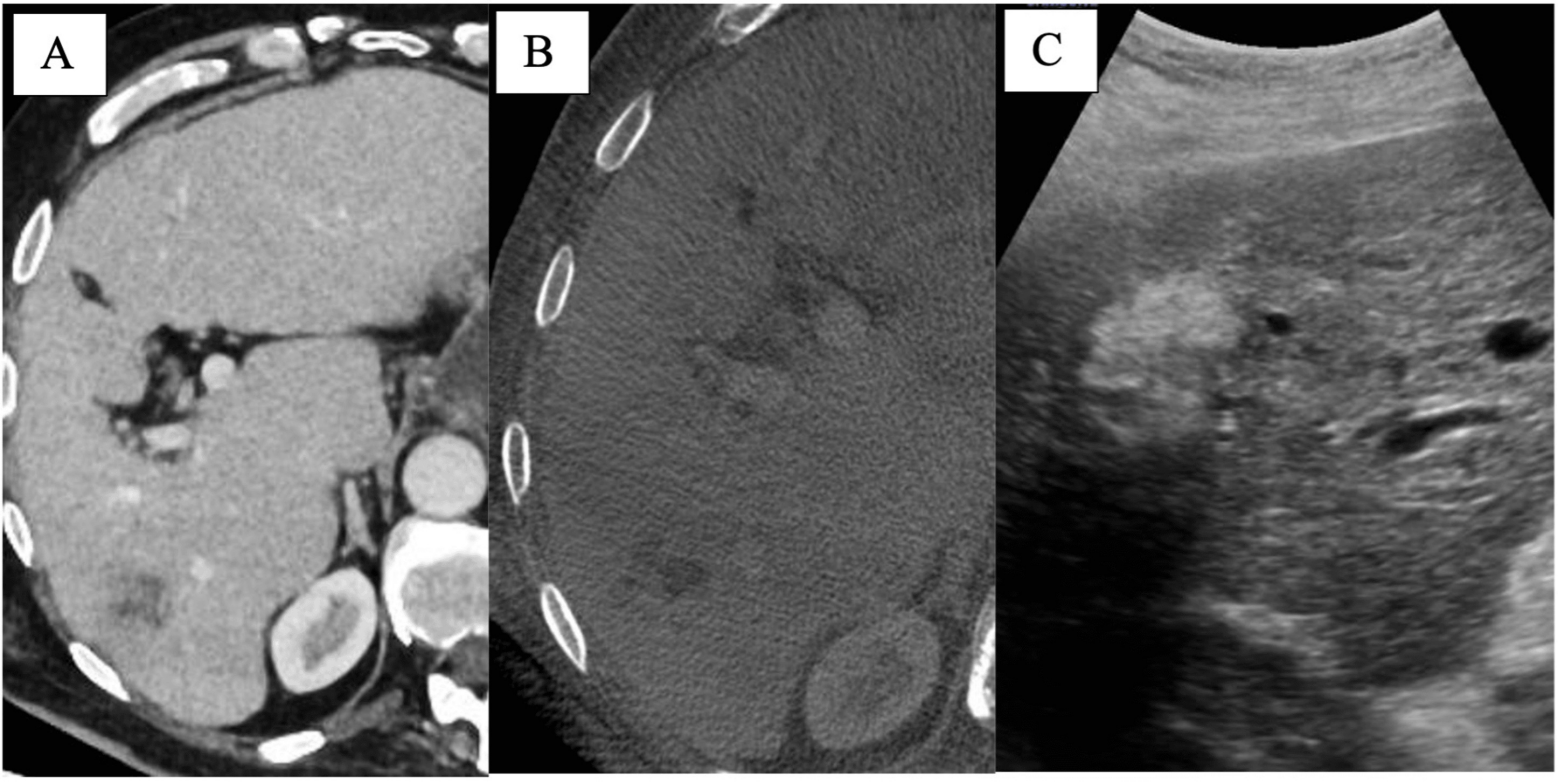
MWA of primary HCC: lesion appearance prior to ablation A single HCC lesion is shown on conventional CT prior to ablation (A). Its appearance on angiographic Dyna-CT is shown in image (B) as well as its appearance on ultrasound prior to probe placement (C). MWA: microwave ablation; HCC: hepatocellular carcinoma; CT: computed tomography; Dyna-CT: dynamic volume computed tomography

Figure 2 shows the expected appearance of the ablation on US and angiographic Dyna-CT/CBCT at the start of the procedure.

**Figure 3:**
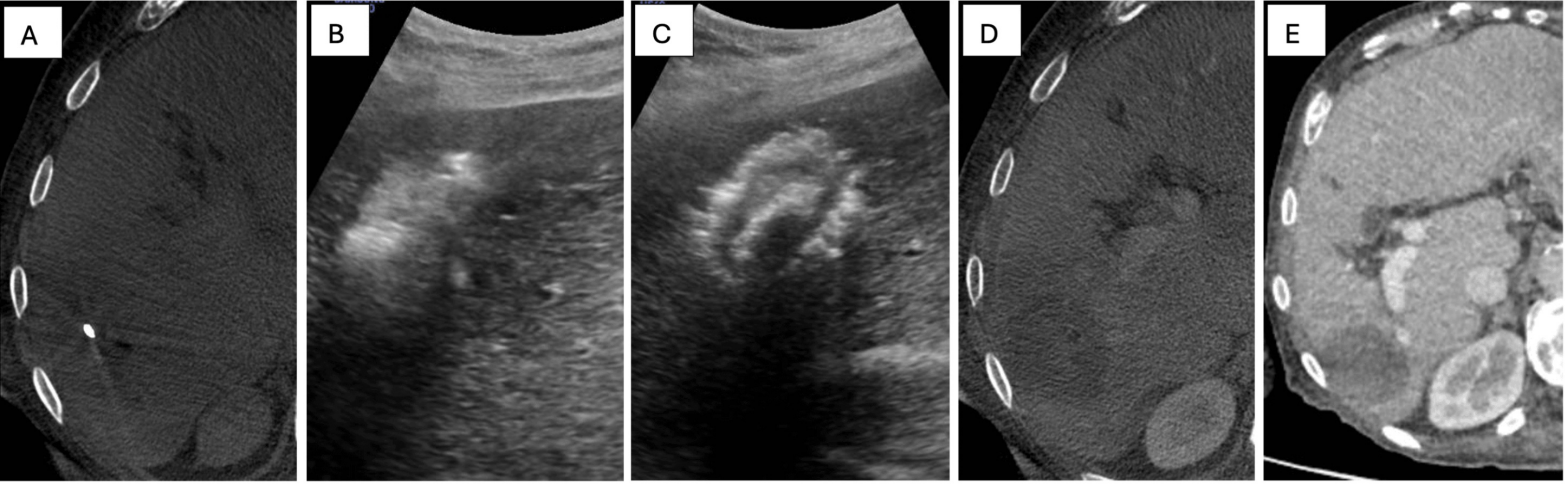
MWA of primary HCC: appearance during and after ablation Intra-procedural and post-procedure appearance of the same HCC lesion as in Figure 2 is shown with the probe at the centre of the lesion on both Dyna-CT and ultrasound. The radio-dense thermal probe is shown within the HCC lesion on Dyna-CT (A). The thermal probe can be seen within the lesion on ultrasound as the microwave is applied producing hyperechoic gas (B). Appearance immediately following ablation is demonstrated on ultrasound with hyperechoic gas at the site of ablation (C). Contrast Dyna-CT shows the immediate post-ablation appearance and confirms adequate coverage (D). Conventional contrast CT demonstrates successful ablation with no recurrence at three months (E). MWA: microwave ablation; HCC: hepatocellular carcinoma; CT: computed tomography; Dyna-CT: dynamic volume computed tomography

Figure 3 shows the same lesion intra-operatively as well as on follow-up contrast-enhanced MDCT.

## Results

All 34 HCC tumour ablation cases performed were included with a total of 42 lesions ablated. In seven patients, more than one lesion was ablated in the same session. The mean lesion size was 20.1 mm±5.92 (range: 10-31 mm). Most lesions were in the right lobe of the liver with only six within segments II and III in the anatomical left lobe (Table 1).

**Table 1:** Demographics and lesion characteristics N: number of patients; SD: standard deviation; mm: millimetres

N	34
Male (%)	24 (70.6)
Mean age (SD)	70.5 (8.21)
Tumour type (%)	Primary HCC (100)
Lesions ablated in the same session (%)	1 lesion: 27 (79.4)
	2 lesions: 6 (17.7)
	3 lesions: 1 (2.9)
Mean largest lesion size: mm (SD) (range)	20.1 (±5.92) (10-31)
Liver segment location (N=42) (%)	II: 3 (7.1)
	III: 3 (7.1)
	IVa: 5 (11.9)
	IVb: 3 (7.1)
	V: 8 (19.1)
	VI: 10 (23.8)
	VII: 2 (4.8)
	VIII: 8 (19.1)

Of the 34 patients treated, 39 (92.9%) lesions had no evidence of disease recurrence on follow-up imaging (Table 2). Three lesions (7.1%) had documented recurrence: two detected between two and six months post-treatment and another at 12 months post-treatment.

**Table 2:** Follow-up data

Follow-up interval	Recurrence (%)
2-6 months (n=26)	2 (7.7)
12 months (n=34)	3 (8.8)

Two patients suffered symptoms of post-ablation syndrome and underwent CT imaging which demonstrated no acute findings (Table 3).

**Table 3:** Complications and corresponding lesion characteristics CT: computed tomography

Age	Sex	Lesion size (mm)	Lesion location	Complication
71	M	25	Seg 5	Pain requiring CT 3 days post-procedure
		20	Seg 5	
		12	Seg 4b	
54	F	15	Seg 4a	Pyrexia and tachycardia 3 days post-procedure

## Discussion

Successful tumour ablation outcomes necessitate good tumour visualisation, thermal probe placement, and evaluation of the ablation zone [4]. 

The utility of combining CBCT with US guidance to plan, implement, modify, and optimise probe positioning during percutaneous tumour ablation has been assessed in a few studies. CBCT has been applied successfully to guide needle placement and exclude immediate post-ablation complications in a small series of renal tumour ablations [7]. A previous study evaluated the use of Dyna-CT in RFA of HCC with reports of 100% technical success rate on five cases [8]. Only two studies [4,5] and one conference abstract [9] described MWA rather than RFA liver ablations: nine and six cases in the former and 12 in the latter with 100% effectiveness of the technique. Indeed, one of these studies demonstrated the feasibility of a combined US and CBCT technique in MWA of very small HCC lesions, less than 2 cm [4].

Our study is the largest series of patients to date, and in contrast to previous studies combining CBCT and US-guided ablation, it does not rely on US-CBCT image fusion or dedicated needle targeting software to achieve intralesional needle placement but instead uses the free-hand or manual technique. Our results are on par with these, demonstrating 100% feasibility and technical success with 100% complete ablation. In addition, our study describes the longest follow-up period for lesions ablated with this technique, where previously published studies do not comment on the rate of recurrence or incomplete ablation beyond two months.

CBCT has been evaluated in the immediate post-procedure and early post-procedural assessment of the ablation zone following US-guided liver tumour RFA and shown to be nearly equivalent to MDCT in detecting the ablative areas and insufficient safety margins after use with US-guided ablation [10].

Peri-procedural CBCT has excellent correlation with 1-2 month post-procedure MDCT in the assessment of the ablation zone providing immediate and reliable assessment of tumour coverage [5].

C-arm technology is a prerequisite of every modern interventional suite especially for oncological hepatic intervention such as selective internal radiation therapy (SIRT) and transarterial chemoembolisation (TACE). Utilising pre-existing IR suite C-arm technology would benefit departments lacking a dedicated interventional CT scanner. Needle navigation software is available on post-processing workstations, integrating multi-planar C-arm fluoroscopic images to provide a navigation path for puncture and thermal probe insertion in real-time, superseding standard CT-guided ablation [11]. 

US is typically used for initial needle placement but not for monitoring due to the production of gas during the procedure. Combining the use of US and CBCT circumvents this issue by visualising the tumour and the ablation zone, thus allowing both optimum probe positioning within the tumour and adequate assessment of the ablation zone. In addition, a combined approach utilising CBCT and US-guided MWA can be effective in typically difficult-to-ablate lesions such as those in the hepatic dome [12] where there is often risk of injury to lung tissue, respiratory motion, and a limited sonic window [13]. 

In addition to the advantage of real-time image guidance for optimal needle placement and immediate assessment of tumour coverage, performing this technique in a dedicated IR suite allows general anaesthesia to be performed easily and intra-procedural complications such as haemorrhage to be treated without logistical delay. 

Besides the retrospective, monocentric nature of this work, a further limitation is that not all our cases were performed with contrast-enhanced Dyna-CT at the initial phase of the adoption of this technique. However, this did not affect technical efficacy. Despite our study including a length of follow-up longer than previous studies, the follow-up for recurrence is 12 months, and further long-term data would be beneficial. Thirdly, the number of patients treated with this technique, although greater than previously reported, remains small. This study is also limited by its single-operator design and the absence of a comparator group, which may introduce operator-related bias and preclude comparative assessment; moreover, radiation dose considerations may limit the broader applicability of the findings.

## Conclusions

Our data builds on previous smaller series and demonstrates the safety and technical success of a combined US and intra-procedural CBCT technique for MWA of HCC. Furthermore, it demonstrates that a free-hand technique, without the need to procure, learn, and apply image fusion software, can produce accurate and safe results in MWA of HCC. In the era of increasing demand for cross-sectional imaging, utilising the IR suite rather than the diagnostic CT scanner facilitates case load flow in busy radiology departments. It is for these reasons that the utilisation of intra-procedural CBCT during US-guided MWA liver HCC ablation merits consideration and inclusion in the greater arsenal of hepatic interventional oncology techniques. 
